# Morphological polymorphism of *Trypanosoma copemani* and description of the genetically diverse *T. vegrandis* sp. nov. from the critically endangered Australian potoroid, the brush-tailed bettong (*Bettongia penicillata* (Gray, 1837))

**DOI:** 10.1186/1756-3305-6-121

**Published:** 2013-04-26

**Authors:** Craig K Thompson, Adriana Botero, Adrian F Wayne, Stephanie S Godfrey, Alan J Lymbery, RC Andrew Thompson

**Affiliations:** 1 School of Veterinary and Life Sciences, Murdoch University, South Street, Western Australia, 6150, Australia; 2 Science Division, Department of Environment and Conservation, Manjimup, WA 6258, Australia; 3 Fish Health Unit and Freshwater Fish Group, School of Veterinary and Life Sciences, Murdoch University, South Street, Western Australia, 6150, Australia

**Keywords:** *Trypanosoma vegrandis*, *T. copemani*, Morphology, PCR, Sequencing, Fluorescence, Woylie, *Bettongia*, *penicillata*

## Abstract

**Background:**

The trypanosome diversity of the Brush-tailed Bettong (*Bettongia penicillata*), known locally as the woylie, has been further investigated. At a species level, woylies are critically endangered and have declined by 90% since 1999. The predation of individuals made more vulnerable by disease is thought to be the primary cause of this decline, but remains to be proven.

**Methods:**

Woylies were sampled from three locations in southern Western Australia. Blood samples were collected and analysed using fluorescence *in situ* hybridization, conventional staining techniques and microscopy. Molecular techniques were also used to confirm morphological observations.

**Results:**

The trypanosomes in the blood of woylies were grouped into three morphologically distinct trypomastigote forms, encompassing two separate species. The larger of the two species, *Trypanosoma copemani* exhibited polymorphic trypomastigote forms, with morphological phenotypes being distinguishable, primarily by the distance between the kinetoplast and nucleus. The second trypanosome species was only 20% of the length of *T. copemani* and is believed to be one of the smallest recorded trypanosome species from mammals. No morphological polymorphism was identified for this genetically diverse second species. We described the trypomastigote morphology of this new, smaller species from the peripheral blood of the woylie and proposed the name *T. vegrandis* sp. nov. Temporal results indicate that during *T. copemani* Phenotype 1 infections, the blood forms remain numerous and are continuously detectable by molecular methodology. In contrast, the trypomastigote forms of *T. copemani* Phenotype 2 appear to decrease in prevalence in the blood to below molecular detectable levels.

**Conclusions:**

Here we report for the first time on the morphological diversity of trypanosomes infecting the woylie and provide the first visual evidence of a mixed infection of both *T. vegrandis* sp. nov and *T. copemani*. We also provide supporting evidence that over time, the intracellular *T. copemani* Phenotype 2 may become localised in the tissues of woylies as the infection progresses from the active acute to chronic phase. As evidence grows, further research will be necessary to investigate whether the morphologically diverse trypanosomes of woylies have impacted on the health of their hosts during recent population declines.

## Background

Trypanosomes are parasitic protozoans (Sarcomastigophora: Kinetoplastida), which cause disease and death in humans and livestock around the world. *Trypanosoma cruzi* (Chagas disease) and *T. brucei* (African sleeping sickness) are collectively responsible for about 63,000 human deaths per year [1], while *T. evansi* (Surra), *T. vivax* (Nagana) and *T. congolense* (Nagana) are all of great economic concern to livestock production in Africa, Asia and South America [2-4].

By contrast, the trypanosomes of wildlife have been poorly studied. There is however some evidence indicating that these parasitic protozoans may be the causative agents behind population declines and extinctions of endangered fauna. For example, it was 100 years after a report of the extinction of endemic native rats on Christmas Island before evidence was presented identifying *T. lewisi* as possibly being influential during the disappearance of the native rats [5,6]. Recent studies have shown that the unintentional introduction of the black rat (*Rattus rattus*) and its fleas infected with *T. lewisi* may have contributed to the extinction of *R. macleari* and possibly *R. nativitatis*[5,7].

**Figure 1:**
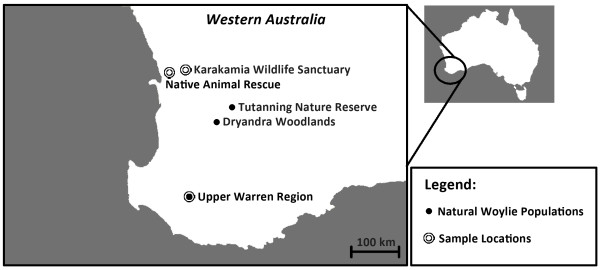
Remaining natural populations of woylies and the sample locations for this study in Western Australia.

It is also possible that trypanosomes have played a potential role during the recent decline of the woylie (*Bettongia penicillata*) in Western Australia (WA) [8,9]. Prior to European settlement of Australia in 1788, woylies were distributed over much of the southern half of mainland Australia [10]. However, as a consequence of human expansion and introduced exotic predators, the natural abundance and distribution of the woylie has been severely challenged [10-12]. By the 1970s woylies became restricted to four small populations located in south-western Australia only; namely Tutanning Nature Reserve, Dryandra Woodlands and the Upper Warren Region (UWR) (which includes the Perup and Kingston populations) [11,13] (Figure 1).

The systematic, broadscale control of foxes using 1080-poison began in the 1970’s and by 1996 the woylie became the first Australian taxon to have its conservation status downgraded from “Endangered” to “Low Risk / Conservation Dependent” (IUCN Red List) because of recovery efforts [11,12]. However, despite the continuous effort to control exotic predators, woylie populations have undergone a further and rapid decline since 1999 [14-16]. The factors responsible for this recent and rapid decline remain unclear, with the future survival of indigenous woylies becoming increasingly uncertain. However, recent spatio-temporal population modelling has hypothesised that disease, in conjunction with predation, may have been the main contributing factors to the recent woylie population declines [17].

During a recent disease investigation, trypanosomes were identified in the blood of woylies from the UWR [9]. The trypanosomes identified were a morphologically distinct species endemic to Australia, which infected between 35% (by molecular techniques) and 43% (by microscopy) of woylies in these declining populations [9]. In an effort to further investigate the influence of trypanosomes upon the recent decline, research was extended to include woylies at the Karakamia Wildlife Sanctuary (KWS) (Figure 1), as this 285 hectare feral-free enclosure contained the only stable high-density sub-population of woylies on mainland Australia [18]. Molecular techniques identified a trypanosome prevalence of 14% at KWS, with microscopy failing to identify any morphological forms in the blood [9]. The observed higher parasite prevalence and parasitaemia (as interpreted by microscopy) in the declining Upper Warren populations helped strengthen the potential role of disease in the recent woylie decline [9].

The present study extends previous work and examines the morphology of the trypanosomes infecting woylies at both the UWR (with eight woylies translocated to NAR as part of a more intensive observational study) and at KWS. We report for the first time on two different morphological phenotypes of *T. copemani* infecting woylies. We also report the first visual identification of a second smaller trypanosome, which is believed to be one of the smallest recorded trypanosomes from mammals. The morphology of this new species has been described and named *T. vegrandis* sp. nov. Also presented is the first visual identification of a mixed infection of both *T. copemani* and *T. vegrandis* sp. nov*.*

## Methods

### Sample collection and preparation

Sheffield traps baited with a mixture of rolled oats, peanut butter and sardines were used to capture woylies from three separate locations in WA. Firstly from the UWR (Figure 1) during November and December, 2010. UWR is predominantly publicly-owned conservation estate and state forest, managed by the Department of Environment and Conservation (DEC), and supports the largest wild woylie population and two of the four indigenous genetically distinct subpopulations extant at the time [13]. Secondly at Native Animal Rescue (NAR) (Figure 1) on seven separate occasions between April 2011 and April 2012. NAR is managed by the Fauna Rehabilitation Foundation, where a predator proof enclosure (110 m x 70 m) has been purpose-built to house 16 woylies. Thirdly at KWS (Figure 1) on two separate occasions during September 2011 and February 2012. KWS is managed by Australian Wildlife Conservancy, where a predator proof fence has been constructed for native Western Australian endangered mammals.

After removing the animal from the trap, a 400 μl sample of blood was collected from the lateral caudal vein using a 25G x 5/8” needle and 1 ml syringe. From the collected blood, 300 μl was placed into a MiniCollect 1 ml EDTA tube (Greiner bio-one, Germany) to prevent clotting and kept at 4°C for DNA extraction and PCR. With the remaining blood, multiple thin blood smears were made from each woylie sampled. Wet mounted slides were also collected during the February 2012 sampling at KWS. After blood collection, woylies were released at the point of capture, except for eight woylies from the UWR, which were translocated to NAR for release.

### Fluorescence *in situ* hybridisation (FISH) and staining

Using *Trypanosoma* Clade B internal forward primer sequence (TVIF [5′- GAC CAA AAA CGT GCA CGT G -3′]) [19], a commercially synthesised probe was manufactured which bound an AlexaFluor350 label at the 5′ end (BioSynthesis, Texas, USA). The AlexaFluro350 label excites at 350 nm and emits at 442 nm.

The FISH protocol used in this study was modified from that developed by Li [20] and was conducted within 24 hours of blood collection. After application of a 125 μl Frame-Seal Incubation Chamber (Bio-Rad, California, USA) to the dried blood slide, cells within the chamber were fixed with 120 μl of 4°C fixative buffer (88 μl of 95% ethanol, 20 μl of deionised H_2_0 and 12 μl of 25x SET buffer [3.75 M NaCl, 25 mM EDTA, 0.5 M Tris HCl @ pH 7.8]). The buffer was left to incubate at 4°C for 40 minutes. The fixative buffer was then drained from the chamber using filter paper and washed with PBS (950 ml of distilled H_2_O, and 50 ml of 20x PBS stock solution [160.0 g/L of NaCl, 24.2 g/L of KH_2_PO4 and 6.8 g/L of K_2_HPO_4_]). After drying the slide in an incubation oven at 58°C for 15 minutes, the cells within the chamber were dehydrated using 50%, 80% and 96% ethanol steps for a period of 90 seconds each, after which, the slide was allowed to air dry.

The remaining steps of the FISH protocol were conducted in a darkened room. To the slide chamber, 125 μl of hybridisation mix (2 μl of TVIF probe (20 μM), 5x SET buffer, Igepal-CA630 [Sigma, Castle Hill, NSW, Australia] and 25 μg/ml polyA potassium salt [Sigma, Castle Hill, NSW, Australia]) was added and the slide placed into an incubation oven at 58°C for 90 minutes. The hybridisation mix was removed from the chamber with filter paper and the cells within the chamber were washed with 1x SET buffer preheated to 58°C. An additional 125 μl of the preheated 1x SET buffer was added to the slide chamber and the slide placed into an incubation oven at 58°C for 15 minutes; this step was repeated once more. The 1x SET buffer was drained out of the chamber with filter paper and the frame-seal removed. The slide was allowed to air dry in the darkened room.

Once dried, the slide was then stained with Modified Wright’s stain in the darkened room and allowed to dry again. Due to an auto-fluorescence issue with anti-fading agents and the Modified Wright’s stain, a drop of distilled water was placed onto the slide and covered with a 50 mm cover slip. The edges of the cover slip were sealed with clear nail-polish to prevent evaporation. The slide was then placed into a dark storage box until viewed under the microscope.

### Microscopy and image acquisition

The hybridised and stained slides were examined with a BX51 microscope (Olympus, Japan) using white light, as well as ultraviolet light (330 – 385 nm) through an emission filter (420 nm). The Alexafluor350 probe fluoresced bright blue under the ultraviolet light conditions. Slides were scanned using the 40x objective lens, with digital images captured using the 100x objective lens. Digital images with an inserted scale bar were captured in a TIFF file format using a microscope mounted camera and DP Controller (Olympus, Japan). Measurements of the key morphological features as described by Hoare [21] were made using Adobe Photoshop CS5 Extended (Adobe Systems Incorporated, USA).

### Morphology

Key morphological measurements were recorded for each of the trypanosomes observed. Morphological traits recorded included total length (L) (including free flagellum), width over the nucleus (W), distance of the posterior to kinetoplast (PK), posterior to nucleus (PN), kinetoplast to nucleus (KN), anterior to nucleus (NA) and length of the free flagellum (FF). For *T. copemani* the kinetoplast-length (K-l) and kinetoplast-width (K-w) were also measured.

Two additional ratios were calculated as they have been used previously to discriminate species of trypanosomes [21]; the Nucleus Index (NI) (=PN/NA) and the Kinetoplast Index (KI) (=PN/KN). When NI=1, the nucleus is in the middle of the body; NI<1, the nucleus is posteriorly located in the body; and N1>1, the nucleus is anteriorly located in the body [21]. When KI=2, the kinetoplast is half way between the posterior and nucleus; KI<2, the kinetoplast is closer to the posterior than to the nucleus; and KI>2, the kinetoplast is closer to the nucleus than the posterior end of the body [21].

Differences in morphology between groups of trypanosomes were tested over all morphological traits (except the two ratio traits) with multivariate analysis of variance (MANOVA), and differences between groups for each trait (including the ratio traits) were tested by single factor analyses of variance, using a Bonferroni correction to maintain an experiment-wide Type I error rate of 5%. If the MANOVA showed a significant difference between groups, then discriminant analysis was used to detect the best combination of traits separating the groups. All statistical analyses were conducted with the software JMP 10.0 [22].

### DNA Extraction

Blood collected in EDTA tubes were used for genomic DNA extraction. DNA was extracted from 300 μl of host blood using the Wizard^®^ Genomic DNA Purification Kit (Cat# A1125) as per the protocol for whole blood extraction (Promega, Wisconsin USA). DNA was eluted in 60 μl of DNA Rehydration Solution and stored at −20°C prior to use. A negative control was included in each batch of DNA extractions, which contained no blood.

### Clade-specific PCR

Three separate clade-specific nested PCR protocols were used to amplify the trypanosome 18S rDNA region using primers and PCR reactions as previously described [19,23]. However, this study used different PCR conditions for four of the primer pairs. For *T. copemani* external primers (S825F and SLIR) [19], the pre-PCR step was 1 cycle of 94°C for 5 mins, 50°C for 2 mins and 72°C for 4 mins, followed by 35 cycles of 94°C for 30 secs, an annealing temperature of 57°C for 30 secs and an extension temperature of 72°C for 2 mins 20 secs, with a final step of 72°C for 7 mins. For *T. copemani* internal primers (WoF and WoR) [23] and Clade B external and internal primers (TVEF, TVER, TVIF, TVIR) [19], the annealing temperature for the 35 cycles was 58°C for 30 secs, while the extension temperature was the same but held for only 50 seconds per cycle. *T. gilletti* species-specific primer sets were used as previously described [23].

Four controls were used in every nested PCR and included the negative control from the DNA extraction, a primary and a secondary PCR negative control and PCR positive control. All were monitored to ensure reliability of results. PCR products were run on a 1.5% agarose gel using SYBR Safe Gel Stain (Invitrogen, California USA) and visualized by illumination with UV light.

### Sequencing PCR

A fourth nested PCR was used to amplify positive samples of *T. copemani* and *T. vegrandis* sp. nov. for sequencing. This technique targeted the 18S rDNA region and used the primers and PCR reaction as previously described [19] but with different PCR conditions for each of the three primer pairs. For the external primers SLF and S762R, the pre-PCR step was 1 cycle of 94°C for 5 mins, 50°C for 2 mins and 72°C for 4 mins, followed by 35 cycles of 94°C for 30 secs, an annealing temperature of 55°C for 30 secs and 72°C for 2 mins 20 secs, with a final step of 72°C for 7 mins. For the first pair of internal primers S823F and S662R, the annealing temperature for the 35 cycles was 56°C for 30 secs, while for the second pair of internal primers S825F and SLIR, the annealing temperature for the 35 cycles was 57°C for 30 secs. The combination of these two PCR products amplified a 1410 bp amplicon for sequencing. Four controls were used as described above. PCR products were run on a 1.5% agarose gel using SYBR Safe Gel Stain (Invitrogen, California USA) and visualized by illumination with UV light.

PCR products of appropriate size were purified using the Agencourt AMPure PCR Purification system (Beckman Coulter, California USA) as per the manufacturer’s instructions and sequenced using an ABI Prism™ Terminator Cycle Sequencing Kit (Applied Bio-systems, California USA) on an Applied Bio-System 3730 DNA Analyser. The resulting sequences were then analysed and aligned using ClustalX 2.1.

## Results

### *Trypanosoma copemani-* microscopy and image acquisition

**Figure 2:**
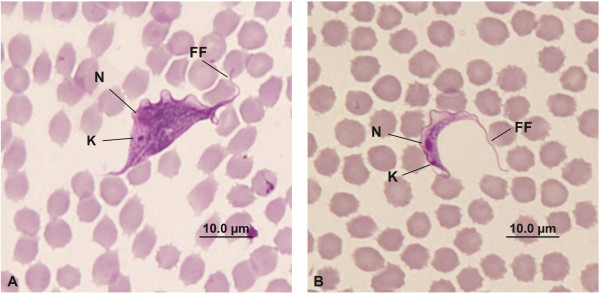
**The different morphological forms of *T. copemani *identified from the same host. A** - Broad trypomastigote form. **B** - Slender trypomastigote form. K= Kinetoplast, N= Nucleus, FF= Free Flagellum.

During the trapping sessions at the UWR, eight of the 15 blood samples examined by microscopy were identified positive for *T. copemani* infections. From these eight infected woylies, a total of 110 trypanosomes were identified in the blood smears. All were trypomastigote forms and their overall measurements are presented in Table 1. The trypomastigotes observed were both broad (N=78) (Figure 2A) and slender (N=32) (Figure 2B). No divisional stages of the trypanosomes were observed in the blood smears. These same eight positive woylies were translocated to the purpose built enclosure at NAR and were part of the temporal study undertaken there.

**Table 1:** **Morphological traits of the trypomastigotes of *T.copemani*: mean measurements (μm) ± standard error (range)**

**Measurements**	**Overall**	**P1**	**P2**
**(μm)**	**(N=110)**	**(N=82)**	**(N=28)**
L	37.34 ± 0.33 (30.25 - 45.19)	36.35 ± 0.35 (30.25 - 45.19)	40.26 ± 0.53 (33.41 - 43.84)
W	6.12 ± 0.18 (1.15 - 10.23)	5.98 ± 0.21 (1.51 - 10.23)	6.53 ± 0.31 (3.71- 9.24)
PK	11.44 ± 0.22 (3.93 - 15.89)	11.49 ± 0.27 (3.93 - 15.89)	11.27 ± 0.35 (5.43 - 14.79)
PN	15.42 ± 0.23 (7.34 - 19.53)	14.98 ± 0.27 (7.34 -19.04)	16.71 ± 0.32 (11.10 - 19.53)
KN	4.36 ± 0.10 (2.52 - 7.31)	3.92 ± 0.07 (2.52 - 5.80)	5.66 ± 0.17 (4.19 - 7.31)
NA	15.85 ± 0.23 (9.30 - 22.06)	15.44 ± 0.26 (9.30 - 22.06)	17.06 ± 0.36 (13.15 - 20.91)
NI	0.98 ± 0.02 (0.39 - 1.42)	0.98 ± 0.02 (0.39 - 1.42)	0.99 ± 0.03 (0.60 - 1.37)
KI	3.64 ± 0.08 (1.38 - 5.96)	3.85 ± 0.09 (1.38 - 5.96)	3.02 ± 0.10 (1.96 - 4.20)
FF	8.24 ± 0.19 (3.44 - 12.39)	8.17 ± 0.22 (3.44 - 12.39)	8.44 ± 0.32 (4.38 - 12.35)
K-l	1.02 ± 0.02 (0.61 - 1.31)	1.06 ± 0.02 (0.76 - 1.31)	0.92 ± 0.04 (0.61 - 1.25)
K-w	0.71 ± 0.01 (0.50 - 1.00)	0.72 ± 0.01 (0.50 - 1.00)	0.69 ± 0.02 (0.50 - 0.93)

**Figure 3:**
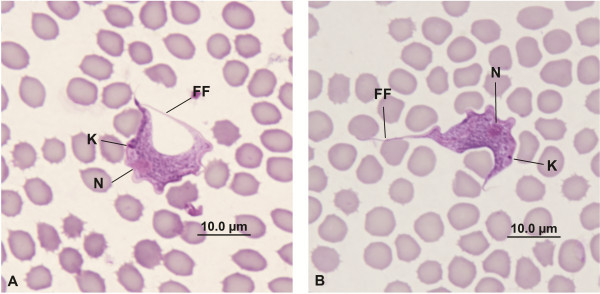
**The different morphological phenotypes of *T. copemani *identified from different hosts. A** - *T. copemani* Phenotype 1 (P1). **B** - *T. copemani* Phenotype 2 (P2). K= Kinetoplast, N= Nucleus, FF= Free Flagellum.

When considering the phylogenetic analysis presented by Botero *et al.,*[19] and the sequencing results below, the 110 trypomastigotes measured were separated into two groups. The first group was “*T. copemani* Phenotype 1” (P1) comprising trypomastigotes found in Woylie ID: WC2741, WC2830, WC2842, WC2844 & WC2920 and the second was “*T. copemani* Phenotype 2” (P2) comprising trypomastigotes found in Woylie ID: WC2807, WC2841 & WC2930. Mean morphological traits for *T. copemani* P1 (Figure 3A) and *T. copemani* P2 (Figure 3B) are shown in Table 1.

From the MANOVA there was a significant morphological difference between groups (F_9,100_ = 22.06, P < 0.0001). Univariate tests found significant differences between groups in KN (F_1,108_ = 131.42), L (F_1,108_ = 34.10), KI (F_1,108_ = 22.73), K-l (F_1,108_ = 13.98), PN (F_1,108_ = 12.11) and NA (F_1,108_ = 10.60) (P < 0.05 for all tests, with the Bonferroni). Discriminant analysis correctly classified 95.5% of the original grouped cases along one canonical discriminant function, which loaded most heavily for KN (0.78), L (0.40), K-l (−0.26), PN (0.24) and NA (0.22).

### *Trypanosoma copemani-* PCR and sequencing

The clade-specific PCR confirmed the presence of *T. copemani* in the eight woylies at the time of translocation from the UWR to NAR. Two distinct genotypes were also identified by sequencing, having a 13 base-pair variation over the larger combined 1410 bp amplicon. The grouping of the eight infected woylies based on the two different genotypes was the same as the phenotypic grouping presented above.

The temporal molecular analysis of infected woylies at NAR between April 2010 and April 2011 indicated that the five woylies infected with *T. copemani* P1 remained PCR positive throughout the study period (Table 2). The November 2011 and April 2012 positive samples were sequenced and confirmed the continued presence of *T. copemani* P1. On the one and only occasion PCR failed to identify the presence of *T. copemani* in these five woylies (WC2842 during Dec 2011; < 50 μl of blood was collected from this woylie on this occasion) microscopy was used alone to confirm the morphological presence of two trypomastigotes on the blood slides.

**Table 2:** **PCR results showing the presence of *T. copemani *P1 and P2 tested at NAR between April 2011 and April 2012**

**Woylie ID**	**Trypanosome**	**April**	**June**	**July**	**Nov**	**Dec**	**March**	**April**
**number**	**species**	**2011**	**2011**	**2011**	**2011**	**2011**	**2012**	**2012**
WC2741	*T. copemani* P1	+	+	+	+	+	+	+
WC2830	*T. copemani* P1	+	+	+	+	+	+	+
WC2842	*T. copemani* P1	+	+	+	+	-*		+
WC2844	*T. copemani* P1		+	+	+	+	+	+
WC2920	*T. copemani* P1	+	+	+	+	+	+	+
WC2807	*T. copemani* P2	+	+	-	+	+	-	-
WC2841	*T. copemani* P2	+	+	+	+	+	+	+
WC2930	*T. copemani* P2	+	+	+	+	+	-	-

[+] PCR positive for *T. copemani.*

[−] PCR negative for *T. copemani.*

[ ] Woylie not trapped that month.

[*] PCR negative but positive to *T. copemani* by microscopy.

In contrast, of the three woylies infected with *T. copemani* P2, two of them (WC2807 and WC2930) lost the blood form of the parasite to below PCR detectable levels in the full 300 μl of blood from March and April 2012 (Table 2). These negative PCR results were supported by the absence of trypomastigotes in the thin blood smears when examined by microscopy. In an effort to strengthen the reliability of these negative results from March and April 2012, nine additional nested PCR reactions were performed on each of the DNA extractions from WC2807 and WC2930. All 40 nested PCR reactions returned a negative result. The November 2011 and April 2012 positive samples were sequenced and confirmed the continued presence of *T. copemani* P2.

### *Trypanosoma vegrandis* sp. nov. *-* microscopy and image acquisition

During sampling at KWS in February 2012, wet blood mounts were made from four woylies previously sampled in September 2011 and shown by PCR to be mono-infected with *T. vegrandis* sp. nov. The movement of this novel motile flagellated trypanosome was observed by microscopy as being a characteristic “cork-screw type” action.

**Figure 4:**
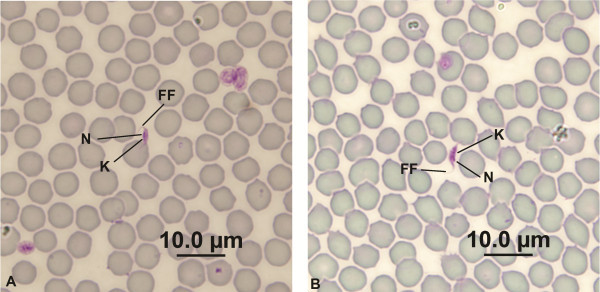
**The morphological form of *T. vegrandis *sp. nov. A** and **B** - Trypomastigote forms. K= Kinetoplast, N= Nucleus, FF= Free Flagellum.

A total of 20 trypanosomes were identified in stained blood smears from these same four woylies (Woylie ID: 7199222, 7236356, 7225370 & K734) and measured. All were identified as trypomastigote forms (Figure 4). The overall measurements of these 20 trypomastigotes are presented in Table 3.

**Table 3:** **Morphological traits of the trypomastigotes of *T. vegrandis *sp. nov.: mean measurements (μm) ± standard error (range)**

**Measurements**	**Overall**	**G1**	**G2**	**Genotype unknown**
**(μm)**	**(N=20)**	**(N=10)**	**(N=10)**	**FISH (N=3)**
L	8.30 ± 0.28 (6.92 - 10.50)	8.12 ± 0.31 (6.92 - 10.25)	8.47 ± 0.47 (7.02 - 10.50)	8.85^*^
W	1.33 ± 0.04 (1.00 – 1.63)	1.33 ± 0.06 (1.04 – 1.63)	1.33 ± 0.05 (1.00 – 1.57)	1.37± 0.03 (1.32 – 1.41)
PK	3.26 ± 0.09 (2.71 - 3.87)	3.30 ± 0.12 (2.78 - 3.87)	3.22 ± 0.14 (2.71 - 3.87)	2.95± 0.12 (2.81 – 3.18)
PN	4.39 ± 0.15 (3.28 – 5.68)	4.28 ± 0.15 (3.28 – 4.78)	4.49 ± 0.26 (3.46 – 5.68)	4.19± 0.16 (3.96 – 4.49)
KN	1.22 ± 0.07 (0.85 – 1.95)	1.11 ± 0.05 (0.95 – 1.39)	1.34 ± 0.12 (0.85 – 1.95)	1.24± 0.05 (1.15 – 1.31)
NA	2.16 ± 0.11 (1.56 – 3.27)	2.02 ± 0.12 (1.56 – 2.87)	2.30 ± 0.17 (1.76 – 3.27)	2.62± 0.10 (2.47 – 2.82)
NI	2.10 ± 0.09 (1.42 – 2.80)	2.18 ± 0.13 (1.59 – 2.80)	2.01 ± 0.13 (1.42 – 2.61)	1.61± 0.11 (1.46 – 1.82)
KI	3.70 ± 0.15 (2.70 – 4.95)	3.92 ± 0.21 (3.24 – 4.94)	3.47 ± 0.19 (2.70 – 4.95)	3.37± 0.06 (3.25 – 3.44)
FF	1.86 ± 0.10 (1.24 – 2.88)	1.83 ± 0.17 (1.28 – 2.88)	1.89 ± 0.13 (1.24 – 2.57)	2.40^*^

* Sample Number = 1.

When considering the phylogenetic analysis presented by Botero *et al.,*[19] and the sequencing PCR results below, the 20 trypomastigotes measured were separated into two groups. The first group was “*T. vegrandis* sp. nov. Genotype 1” (G1), comprising trypomastigotes found from Woylie ID: 7199222 & 7236356 and the second was “*T. vegrandis* sp. nov. Genotype 2” (G2) comprising trypomastigotes found in Woylie ID: 7225370 & K734. Mean morphological traits for each group are shown in Table 3. There were no significant differences between groups using all morphological traits in MANOVA (F_7,12_ = 0.97, P = 0.49) or using each trait separately in univariate ANOVA’s. This genetically varied clade appears to be represented by a single, morphologically unique phenotype (Figure 4).

**Figure 5:**
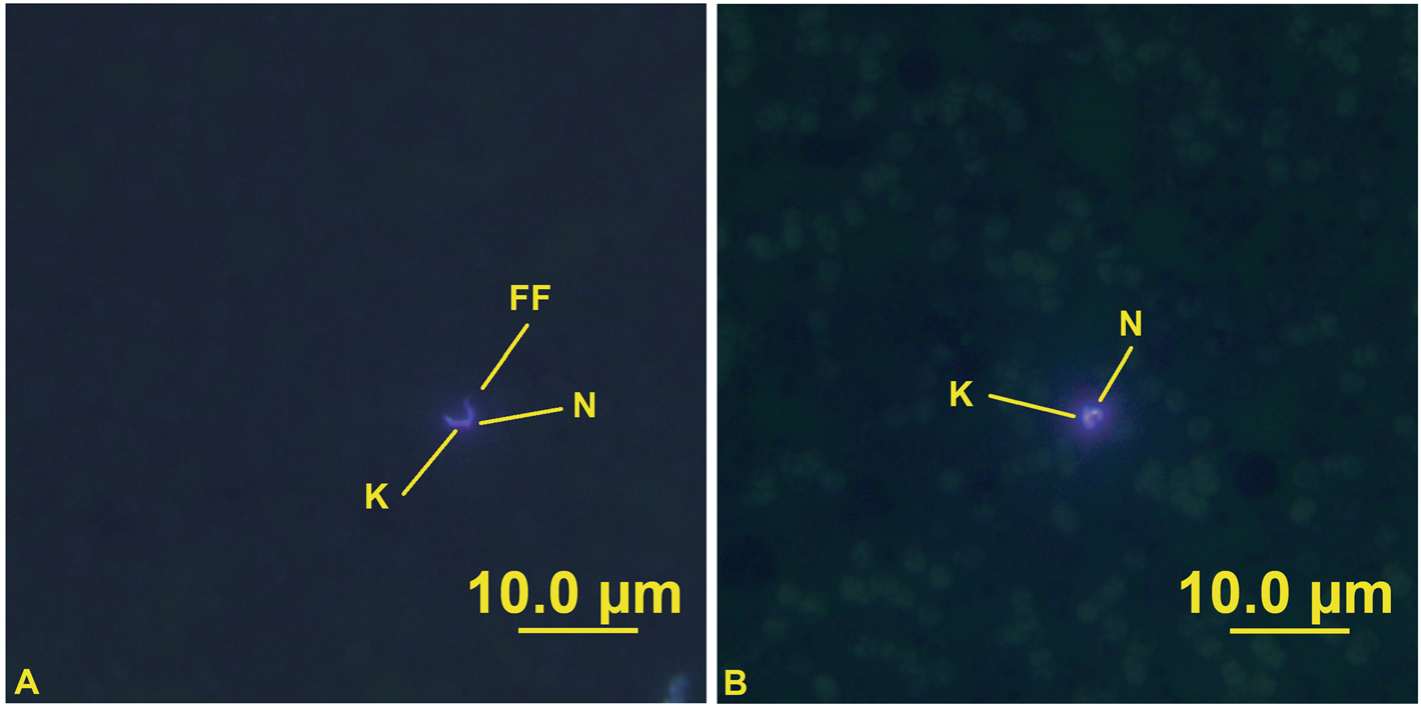
**Fluorescence in situ hybridisation of *T. vegrandis *sp. nov. A** and **B** - Fluorescent trypomastigote forms. K= Kinetoplast, N= Nucleus, FF= Free Flagellum.

During the April 2012 sampling at NAR, blood mounts from one woylie known to be PCR positive for infections with *T. vegrandis* sp. nov. (WC2741; Table 4) were successfully hybridised and stained. Three trypomastigotes were identified by the hybridisation of the fluorescence probe *in situ* (Figure 5). The morphological measurements of these three trypomastigotes are presented in Table 3.

**Table 4:** **PCR results showing the presence of *T. vegrandis *sp. nov. tested at NAR between April 2011 and April 2012**

**Woylie ID number**	**Trypanosome species**	**April 2011**	**June 2011**	**July**	**Nov**	**Dec**	**March**	**April**
				**2011**	**2011**	**2011**	**2012**	**2012**
WC2741	*T. vegrandis* sp. nov.	+	+	+	+	+	+	+
WC2830	*T. vegrandis* sp. nov.	-	-	-	-	-	-	-
WC2842	*T. vegrandis* sp. nov.	-	-	-	-	-		-
WC2844	*T. vegrandis* sp. nov.		+	+	+	+	+	+
WC2920	*T. vegrandis* sp. nov.	+	+	+	-	-	+	+
WC2807	*T. vegrandis* sp. nov.	-	-	-	-	-	-	-
WC2841	*T. vegrandis* sp. nov.	-	-	-	-	+	-	-
WC2930	*T. vegrandis* sp. nov.	-	-	-	-	-	-	-

[+] PCR positive for *T. vegrandis* sp. nov.

[−] PCR negative for *T. vegrandis* sp. nov.

[ ] Woylie not trapped that month.

### *Trypanosoma vegrandis* sp. nov. *-* PCR and sequencing

The clade-specific nested PCR confirmed the presence of *T. vegrandis* sp. nov. in four woylies at KWS (Woylie ID: 7199222, 7236356, 7225370 & K734) during the trapping sessions of September 2011 and February 2012. These same four woylies tested negative to *T. copemani* and *T. gilletti*. Two distinct genotypes were identified by the sequencing of the PCR products, the first group was “*T. vegrandis* sp. nov. G1”, again comprising trypomastigotes found in Woylie ID: 7199222 & 7236356 (representing G3 and G6 genetic sequences of Clade B [19]). The second group was “*T. vegrandis* sp. nov. G2”, again comprising trypomastigotes found in Woylie ID: 7225370 & K734 (representing G4, G5 and G7 genetic sequences of Clade B [19]).

Of the five *T. copemani* P1 positive woylies at NAR, three of them (WC2741, WC2844 and WC2920) were also PCR positive for *T. vegrandis* sp. nov*.* Both WC2741 and WC2844 maintained a consistent mixed infection during the 13 month sampling period (Table 4). Of the three woylies infected with *T. copemani* P2, there was only one occasion that *T. vegrandis* sp. nov. was detected by the species-specific PCR as a mixed infection within the blood; this being WC2841 in December 2011 (Table 4).

All attempts to amplify *T. vegrandis* sp. nov. in the mixed presence of *T. copemani* using the sequencing PCR protocol failed. Also all attempts to amplify *T. vegrandis* sp. nov. with the *T. gilletti* species-specific PCR protocol failed.

### Description of a new species- *Trypanosoma vegrandis* sp. nov

Based on results presented here we propose the name *Trypanosoma vegrandis* sp. nov. for this morphologically and genetically distinct species found within the woylie.

*Diagnosis:* Morphological analysis of various blood forms from the woylie or Brush-tailed Bettong (*B. penicillata*) including microscopy of live motile bodies, fluorescence *in situ* hybridisation and Modified Wright’s staining of fixed trypomastigotes. Description represents a single phenotype encompassing two different genotypes identified by phylogenetic analysis of the 18S rDNA and gGAPDH gene [19].

The trypomastigotes of *T. vegrandis* sp. nov. have a curved body which is drawn out to a pointed posterior end. They are a small trypanosome with a smallest recorded length being 6.92 μm. The width of the trypanosome is about 16% that of its total length and the free flagellum is relatively long, being over 20% of the total length. The nucleus is located in the anterior half of the body, with the posterior kinetoplast positioned closer to the nucleus than to the posterior edge of the body. The distance between the nucleus and kinetoplast is about 20% of the body length, when excluding the free flagellum.

The mean total length of the trypomastigotes found in the blood of the woylie was 8.30 μm (range: 6.92 - 10.50 μm), mean width- 1.33 μm (range: 1.00 - 1.63 μm), mean posterior edge to kinetoplast distance- 3.26 μm (range: 2.71 - 3.87 μm), mean posterior edge to nucleus distance- 4.39 μm (range: 3.28 - 5.68 μm), mean kinetoplast to nucleus distance- 1.22 μm (range: 0.85 - 1.95 μm), mean nucleus to anterior edge distance- 2.16 μm (range: 1.56 - 3.27 μm) and mean free flagellum length- 1.86 μm (range: 1.24 - 2.88 μm). The mean NI index was 2.10 μm (range: 1.42 - 2.80 μm) and mean KI index was 3.70 μm (range: 2.07 - 4.95 μm).

#### Taxonomic summary

##### Vertebrate type host

Brush-tailed Bettong (*Bettongia penicillata*)

##### Vertebrate additional hosts

Western Grey Kangaroo, Quenda, Tammar Wallaby, Chuditch [19]

##### Invertebrate vector

Unknown

##### Morphological type location

Karakamia Wildlife Sanctuary (S31.82073; E116.24604)

##### Additional locations

Upper Warren Region (S34.11528; E116.32362), Native Animal Rescue (S31.86677; E115.89072) and Dwellingup, WA

##### Site of infection

Blood, brain (results not shown) as well as skeletal muscle, heart, lung, oesophagus, tongue, kidney, bone marrow, liver and spleen [19]

##### Pre-patent and patent periods

Unknown

##### Subacute phase

Unknown

##### Chronic phase

Unknown

##### Etymology

This species has been given the name *vegrandis* due to the small size; vegrandis is a logical name as it means diminutive, small and tiny.

### *Mixed Infection-* microscopy and image acquisition

**Figure 6:**
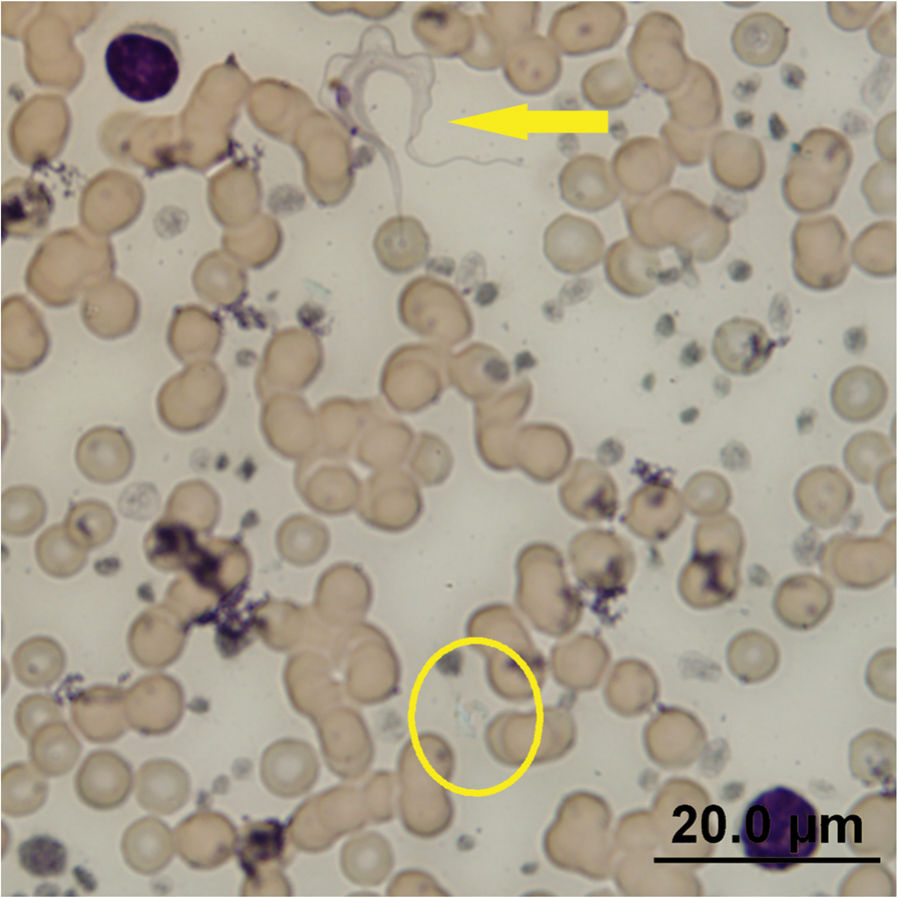
**Trypomastigotes of *T. copemani *P1 (arrow) and *T. vegrandis *(circle) from NAR.**

The mixed infection of *T. copemani P1* and *T. vegrandis* identified by molecular methodology for Woylie ID: WC2741 at NAR was confirmed by microscopy in April 2012 during the hybridization and staining procedure. When the fluorescent conditions of Figure 5B were changed to white light microscopy, the field of view contained not only the hybridised *T. vegrandis* (Figure 6- circle) but also contained the thin trypomastigote of *T. copemani* P1 (Figure 6- arrow). Figures 4 and 6 also illustrate the varying translucency of the *T. vegrandis* trypomastigote forms when stained. The absence of fluorescence in the vicinity of *T. copemani* in Figure 5B confirmed the specificity of the probe.

## Discussion

Overall, *T. copemani* trypomastigotes in the blood of woylies were characterised by a long curved body. The nucleus, on average, was near the centre of the body and the posterior, oval-shaped kinetoplast was positioned closer to the nucleus than to the posterior edge of the body. A free flagellum was present and was about 20% of the total length. The two phenotypes of *T. copemani* were distinguished by the statistically significant differences in a number of morphological traits, in particular the distance between the nucleus and kinetoplast. The KN distance for P1 was 3.92 ± 0.07 μm and for P2 was 5.66 ± 0.17 μm. The different KN distances were not believed to be an artefact as all blood smears were made in exactly the same manner and the phenotypic grouping of the eight woylies at NAR matched exactly with the genotypic grouping of these same woylies.

From the *T. copemani* measurements, we suggest that the different thicknesses observed represent different life stages of the trypanosome within the host, as both broad and slender forms were observed simultaneously within individual P1 and P2 infected hosts. We believe that the broad trypomastigote was the blood form responsible for the reproductive phase (71% of trypomastigotes measured) and the slender form was the adult trypomastigote. Similar size variations of the trypomastigote blood forms have been observed in various mammalian hosts and includes *T. lewisi, T. musculi, T. evotomys* and *T. zapi*[21]. Varying trypomastigote thickness was also observed in the Gilbert’s Potoroo (*Potorous gilbertii)*, with *T. copemani* trypomastigotes grouped as slender, medium and broad [24].

There appears to be host-induced morphological variation of trypanosomes found infecting wildlife. The morphology of *T. copemani* observed in the woylie, for example, differs to that of *T. copemani* found in Gilbert’s potoroo, being relatively longer and thinner. The smallest length recorded in the woylie was 30.25 μm, while in the potoroo it was 25.0 μm and the widest length recorded in the woylie was 10.23 μm, while in the potoroo it was 15.4 μm [24]. Other dissimilarities included a larger PK mean in the woylie (11.44 μm compared to 8.1 μm in the potoroo), a smaller KN and FF mean in the woylie (4.36 μm and 8.24 μm compared to 5.8 μm and 10.8 μm respectively in the potoroo) [24]. Also dividing trypomastigotes of *T. copemani* were identified in the potoroo [24], whereas we failed to locate any divisional forms in the woylies. Compared to *T. copemani* in the quokka (*Setonix brachyurus*), trypomastigotes of *T. copemani* in the woylie were wider (6.16 μm compared to 4.2 μm in the quokka), had a larger PK and NA mean (11.44 μm and 15.85 μm compared to 6.5 μm and 13.7 μm respectively in the quokka) and a smaller KN and FF mean (4.36 μm and 8.24 μm compared to 5.9 μm and 12.1 μm respectively in the quokka) [24]. This polymorphism emphasises the importance of using both morphological and genetic criteria in describing trypanosomes from wildlife.

The two morphological phenotypes of *T. copemani* described in the present study, P1 and P2, correspond to Clade A Genotypes 1 and 2 respectively, of which only P2 (≈ G2) has the ability to invade and divide within the cells of the host [19]. Two woylies, which were sampled over time at NAR (WC2807 and WC2930), appeared to lose their infection with *T. copemani* P2; this was based on the lack of PCR detection of trypanosomes in the peripheral blood. This may be because the infection became localised to the tissues of the host. In contrast, woylies infected with *T. copemani* P1, including those concurrently infected with *T. vegrandis* consistently maintained detectable levels of *T. copemani* P1 during this study. Due to our small samples sizes for these observations, further work is required to determine if *T. copemani* P1 is capable of infecting tissues, which may indicate a significant difference in virulence potential between the two forms of *T. copemani*.

The chronic effect of *T. copemani* P2 within the woylie remains unknown. However, it has been hypothesised from histopathological observations that when *T. copemani* P2 invade host cells as part of the life cycle in the woylie, it may initiate a strong inflammatory response of the host, with significant tissue degeneration occurring in the heart, oesophagus, kidney and tongue [19]. The pathological lesions and tissue degeneration observed within infected woylies with *T. copemani* P2 show similarities to the pathological changes observed in infected opossums with *T. cruzi*[19,25]. Overtime these pathological changes to the woylies may reduce its fitness and be a contributing factor during its recent decline.

The naming of *T. vegrandis* was supported by microscopic visualisation of live motile stages, Modified Wright’s stained trypomastigotes and hybridised forms stained with a species-specific fluorescent probe. It is also complemented by the genetic amplification using species-specific Clade B primers, the failure of the *T. vegrandis* species-specific fluorescent probe to hybridise to *T. copemani* (Figure 5B &6), and failure of genetic amplification using the *T. gilletti* species-specific primers.

In spite of the genetic variability seen in the phylogenetic analysis of Clade B by Botero *et al.,*[19] we observed morphological uniformity, grouping the genetic sequences of Clade B G3 – G7 together as a single morphological phenotype. This provides further support that the description of a trypanosome species should not be based on morphology alone, due to the polymorphic nature of the trypomastigotes in the blood (as discussed above with *T. copemani* in different hosts). As such, further studies are required to determine whether *T. gilletti* has morphological affinities with *T. vegrandis*, since *T. gilletti* was described solely on genetic data [26].

The morphology of *T. vegrandis* has been elusive since its molecular identification and in hindsight, is not surprising that it was not detected in previous studies when its small size and translucent nature are taken into account. We now know that *T. vegrandis* is the most prevalent trypanosome within this sub-population of woylies at KWS (unpublished data) but may well have been overlooked in previous surveys at KWS [9] and other locations [8,27], because of the limitations of the molecular tools used at the time.

Another reason for the absence of previous morphological observations may be the critical timing between blood collection and fixation of the slide. We believe that the slides need to be fixed and stained within the 24 hour period after blood collection. Increasing the time for fixation of the blood slide results in a degradation of *T. vegrandis*, to a point where it is no longer detectable by microscopy (unpublished data). This was the case for samples collected at KWS in September 2011 where 22 PCR-positive blood smears were collected, and were fixed and stained four weeks later. By this time no morphological forms of *T. vegrandis* were identified by microscopy. It is therefore possible that this may have been the case for the molecular-based reports of *T. gilletti* from koalas and *T. gilletti*-like trypanosomes from woylies where no morphological forms were identified [26,27].

**Figure 7:**
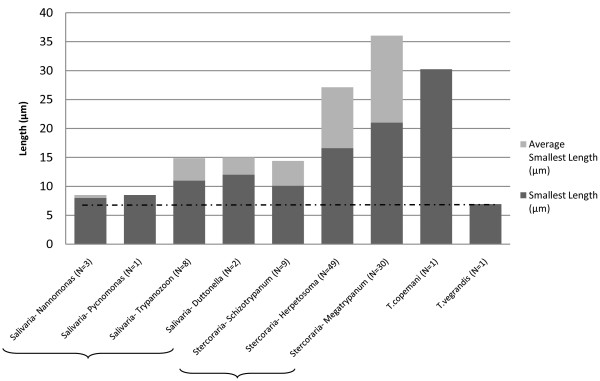
**Smallest reported length and average smallest length of the species within each subgenus of *Trypanosoma***[21], ***T. copemani *and *T. vergrandis.***

Overall, the trypomastigotes of *T. vegrandis* in the blood of woylies were approximately 20% the length of *T. copemani,* with a minimum length of 6.92 μm. The previous smallest reported individual trypomastigote lengths that we found were for *T. congolense* and *T. simiae*, both with a minimum length of 8 μm [21]. *T. vegrandis* is believed to be one of the smallest trypanosomes reported infecting mammals [21,24,26,28-37]. Using the smallest length of both *T. vegrandis* and *T. copemani* from this study and the comprehensive analysis of trypanosome species complied by Hoare [21], Figure 7 compares the smallest individual recorded length for each sub-genus, as well as the mean of the smallest recorded lengths of the species within each sub-genus (N=104).

*T. copemani* was grouped within the subgenus *Herpetosoma* due to the long free flagellum, oval kinetoplast, and the relatively large distance between the kinetoplast and nucleus [24]. Surprisingly, similar morphological ratios of *T. vegrandis* are reported here. Apart from the very small size of *T. vegrandis*, all of the body proportions (except for the NI index) are similar to that of *T. copemani*, with a relatively long free flagellum and a relatively large distance between the kinetoplast and nucleus. At this stage it is very difficult to assign *T. vegrandis* to a subgenus. The phylogenetic analysis of the 18S rDNA and *gGAPDH* genes suggest that *T. vegrandis* may be part of the Stercoraria grouping of trypanosomes as it shares a close evolutionary relationship with *T. copemani* and *T. pestanai* (both being part of the Stercoraria group) [19,21]. Further work is required to understand the transmission dynamics from vector to host, along with the life cycle of the trypanosome in the vector before this can be commented on further.

## Conclusion

In this report we describe the morphological polymorphism of *T. copemani*, which includes the different trypomastigote phenotypes from the blood of woylies. We also provide the first morphological observations and taxonomic description of trypanosomes from a new genetically diverse clade, for which we propose the name *T. vegrandis*. Up until now this small trypanosome has only been identified by PCR from a variety of hosts, including the woylie. Using fluorescence *in situ* hybridisation and light microscopy, we described a mixed trypanosome infection in a woylie, with both *T. copemani* and *T. vegrandis* observed. The temporal reduction of *T. copemani* P2 in the peripheral blood of the woylie and its ability to invade cells may suggest that this more virulent phenotype could become localised within the tissues of the host. Over time, tissue degeneration of the host could result in an overall reduced fitness, making the woylie more susceptible to predation in the wild.

## Competing interests

The author(s) declare that they have no competing interests.

## Authors’ contributions

CKT, AB, AFW and RCAT designed the study; CKT, AFW, SSG, AJL and RCAT implemented the study; CKT managed the data; CKT, AB, SSG and AJL analysed and interpreted the data; CKT wrote the paper. CKT, AFW, SSG and RCAT supervised the different phases of the study. All authors read, revised and approved the final manuscript.
